# IGN004 is an antibody-interferon-alpha fusion protein against a novel tumor-associated antigen with both direct anti-tumor and immunostimulatory effects

**DOI:** 10.1186/2051-1426-3-S2-P245

**Published:** 2015-11-04

**Authors:** Kristopher K Steward, Raj K Sachdev, Michael J Gresser, Sanjay D Khare

**Affiliations:** 1 ImmunGene, Inc., Camarillo, CA, USA

## Background

Antibody-interferon-alpha (IFNα) fusion proteins represent a cancer therapeutic with properties of an antibody-drug conjugate and an immunotherapy, having both direct anti-tumor and immune-activating effects. We report the anti-tumor activity of IGN004, an antibody-IFNα fusion protein against a novel tumor-associated antigen expressed by many solid and liquid tumors.

## Methods

IGN004 was evaluated against a panel of human non-small cell lung cancer (NSCLC), melanoma, multiple myeloma (MM), and AML cell lines. Binding was assessed by flow cytometry and immunohistochemistry (IHC). Anti-proliferative activity and T cell killing of tumor cells by TALL-104 effector cells were assessed by MTS assay. Human tumor xenografts were grown in immunodeficient mice.

## Results

IGN004 unfused antibody bound to the majority of tumor cell lines and primary tumors assessed. Against tumor antigen-positive cells in anti-proliferation experiments, IGN004 demonstrated enhanced potency compared to unfused IFNα while reduced potency was observed in cells lacking antigen expression. IGN004 treatment upregulated MHC class I, PD-L1, and OX-40L on tumor cells. In an *in vitro* T cell killing assay using TALL-104 cells as effectors and A549 NSCLC cells as targets, the addition of IGN004 led to enhanced effector cell killing of tumor (69.2% killing without IGN004 vs. 100% killing with IGN004; p = 0.001). Importantly, IGN004 demonstrated robust *in vivo* efficacy against MM, NSCLC, AML, and melanoma xenografts, including patient-derived xenografts (PDX). Against U266 MM xenografts, IGN004 fusion protein caused complete regression of all tumors and achieved long-term survival in 62.5% of mice. Efficacy was tested against a panel of 14 NSCLC PDX tumors and IGN004 had a response rate of 64%, including tumor regression in 29%. In an AML PDX model, IGN004 treatment caused a reduction in AML cells in the blood, spleen and bone marrow. Against a PDX model of melanoma, IGN004 unfused antibody was ineffective while IGN004 fusion protein inhibited tumor growth.

## Conclusions

IGN004 demonstrated robust anti-tumor activity against both solid and liquid tumors. Targeting of IFNα to the tumor cell surface via antibody resulted in enhanced potency of growth inhibition. The relative IFNα bioactivity is reduced against cells that do not express the target antigen, which may result in a broader therapeutic index. IGN004 demonstrated the ability to enhance the effector T cell mediated killing of NSCLC cells in an *in vitro* assay. Against human xenograft tumors, including PDX, IGN004 had robust *in vivo* anti-tumor efficacy. These results support the further development of IGN004 as a targeted cancer immunotherapy.

